# Ten-kilohertz two-photon microscopy imaging of single-cell dendritic activity and hemodynamics *in vivo*

**DOI:** 10.1117/1.NPh.10.2.025006

**Published:** 2023-05-03

**Authors:** Ruijie Li, Sibo Wang, Jing Lyu, Ke Chen, Xiaxin Sun, Junjie Huang, Pei Sun, Susu Liang, Min Li, Mengke Yang, Hongbang Liu, Shaoqun Zeng, Xiaowei Chen, Longhui Li, Hongbo Jia, Zhenqiao Zhou

**Affiliations:** a Guangxi University, Advanced Institute for Brain and Intelligence, School of Physical Science and Technology, Nanning, China; b Third Military Medical University, Brain Research Center, State Key Laboratory of Trauma, Burns, and Combined Injury, Chongqing, China; c Chinese Academy of Sciences, Suzhou Institute of Biomedical Engineering and Technology, Brain Research Instrument Innovation Center, Suzhou, China; d Sichuan Provincial People’s Hospital, University of Electronic Science and Technology of China, Medical School, Chengdu, China; e Chongqing University, School of Medicine, Center for Neurointelligence, Chongqing, China; f Huazhong University of Science and Technology, Britton Chance Center for Biomedical Photonics, Wuhan National Laboratory for Optoelectronics, Wuhan, China; g Chongqing Institute for Brain and Intelligence, Guangyang Bay Laboratory, Chongqing, China; h Leibniz Institute for Neurobiology, Magdeburg, Germany; i Technical University Munich, Institute of Neuroscience and the SyNergy Cluster, Munich, Germany

**Keywords:** two-photon microscopy, acousto-optic deflector, neural activity, hemodynamics

## Abstract

**Significance:**

The studying of rapid neuronal signaling across large spatial scales in intact, living brains requires both high temporal resolution and versatility of the measurement device.

**Aim:**

We introduce a high-speed two-photon microscope based on a custom-built acousto-optic deflector (AOD). This microscope has a maximum line scan frequency of 400 kHz and a maximum frame rate of 10,000 frames per second (fps) at 250×40  pixels. For stepwise magnification from population view to subcellular view with high spatial and temporal resolution, we combined the AOD with resonance-galvo (RS) scanning.

**Approach:**

With this combinatorial device that supports both large-view navigation and small-view high-speed imaging, we measured dendritic calcium propagation velocity and the velocity of single red blood cells (RBCs).

**Results:**

We measured dendritic calcium propagation velocity (80/62.5−116.7  μm/ms) in OGB-1-labeled single cortical neurons in mice *in vivo*. To benchmark the spatial precision and detection sensitivity of measurement *in vivo*, we also visualized the trajectories of single RBCs and found that their movement speed follows Poiseuille’s law of laminar flow.

**Conclusions:**

This proof-of-concept methodological development shows that the combination of AOD and RS scanning two-photon microscopy provides both versatility and precision for quantitative analysis of single neuronal activities and hemodynamics *in vivo*.

## 1 Introduction

Multiphoton microscopy is currently among the most suitable methods for *in vivo* imaging due to its high tissue penetration depth and submicron spatial resolution,^1^^–^^3^ especially for neuroscience and hemodynamic applications.^4^^–^^10^ In recent years, major innovations in different features of multiphoton microscopy have driven its broader adoption, including larger fields of view,^11^^–^^13^ faster speed,^14^^–^^26^ and deeper imaging depth.^27^^,^^28^ In particular, a series of advances have increased the full-frame two-dimensional (2D) imaging speed of two-photon microscopes to thousands of frames per second (fps),^19^^–^^26^ enabling the capture and recording of neural activities at high temporal resolution without biases in random access coordinate assignments within the image frame. For example, scanned line angular projection microscopy scans line foci along four different axes, analogous to computed tomography, achieving a high frame rate of 1016 Hz, facilitating the imaging of visually evoked glutamate signaling in cortical dendrites *in vivo*.^25^ Wu et al. used another recently developed technique, free-space angular-chirp-enhanced delay, to generate an array of spatiotemporally multiplexed beam-lets, resulting in a high fast-axis frequency of 1 MHz and a frame rate of 1 to 3 kHz. This method was then used for *in vivo* imaging with the genetically encoded voltage indicator, ASAP3.^22^^,^^24^ Zhang et al.^21^ also obtained a full-frame imaging speed of 1 kHz through the multifocal method, which was then used to measure cell flow velocity in cerebral arterioles. Xiao et al.^26^ developed a scan multiplier unit (SMU) to convert a single line scan into a 2D scan of one frame. By combining SMU with a resonance scanner, they achieved a frame rate of 16 kHz, the highest frame rate reported in multiphoton imaging to date.^26^ However, the SMU uses a lenslet array for scan conversion that is not easily switched during a single experiment, consequently limiting changes in scanning parameters.

In the other methods mentioned above, a galvanometer (Galvo) is used as the slow axis scanner, limiting improvements in frame rate to ∼3  kHz due to the mechanical inertia of Galvo.^20^^,^^22^^–^^25^ This limitation has prevented further increases in the temporal resolution of two-photon full-frame planar imaging.

To overcome these obstacles, in this study, we developed a high-speed two-photon microscope that uses an acousto-optic deflector (AOD) with high acoustic velocity to significantly increase line scan frequency to 400 kHz, a ∼fivefold improvement over that in similar previous work,^20^ while incorporating a second AOD for slow axis scanning to expand the maximum frame rate to 10 kHz (@ 250×40  pixels) with 0.1-ms time resolution. By switching the speed compensated cylindrical lens (CYL), the user can switch among a variety of scanning settings. The extremely high imaging speed is essential for the recording the submillisecond scale of neural activity. Here, our proof-of-concept study demonstrates its use in measuring dendritic ${\mathrm{Ca}}^{2+}$ signal propagation *in vivo*, as well as high-speed imaging analysis of red blood cell (RBC) flow in mice, illustrating its capabilities and potentially broad application for high resolution, temporally resolved, subcellular investigations of highly complex neural and vascular systems.

## 2 Methods

### 2.1 Design of AODs

The scanning frequency of an AOD is limited by transit time (T), which is the ratio of the aperture (H) to the sound velocity (v) of the acousto-optic crystal,^29^ as shown in Eq. (1):

$$T=\frac{H}{v}.$$ (1)

In previous studies,^14^^–^^18^^,^^20^ higher resolution points or large fields of view were generally accomplished by selecting AODs with a sound speed of 650 to 820  m/s, with a typical transit time of 3 to 30  μs, consequently limiting scanning frequency to 10 to 80 kHz. Here, we used a high acoustic velocity (v=4200  m/s) AOD (custom-designed, manufactured by Isomet) that relies on the fast acoustic mode of a ${\mathrm{TeO}}_{2}$ crystal for the fast-axis scanner. This AOD has a $4\times 4\;{\mathrm{mm}}^{2}$ (H=4  mm) square aperture, with a ∼1-μs transit time. The shortest line scan period was set to 2.5  μs, resulting in a maximum line scanning frequency of 400 kHz. It should be noted that this is the highest line scanning frequency for an AOD in two-photon imaging applications reported to date.^20^

The use of a high sound velocity AOD increases both line scan frequency and frame rate. However, high sound velocity (v) and a large square aperture (H) usually reduce the acousto-optic quality factor (Q) and diffractive efficiency (η),^30^ as shown in Eq. (2)

$$\eta \propto QP\frac{L}{H}=\frac{n^{6}p^{2}}{\rho v^{3}}P\frac{L}{H},$$ (2)

where n is the refractive index, p is the acousto-optic coefficient, ρ is the crystal density, and L is the acousto-optic interaction length. To address this issue, we maintained diffraction efficiency by increasing ultrasonic power (P) using four phased array transducers to provide adequate ultrasound power (1.5 W each, 6 W total) to the fast-axis AOD, resulting in a diffractive efficiency of ∼50%. To avoid locally excessive temperatures in the acousto-optic crystal, an array transducer design was applied that could disperse heat generated by ultrasound. A water-cooled circulating system was also added to further dissipate heat in the crystal. In addition, the phased array transducers enabled ultrasonic steering to enlarge the bandwidth Δf of the fast-axis AOD and flatten the diffractive efficiency across the bandwidth range.^30^ In this case, the Δf at 1.5 dB bandwidth was ∼50  MHz for the fast-axis AOD.

To maximize the imaging frame rate, the slow-axis scanner also required a relatively high scanning frequency. We, therefore, selected another AOD (4150, GOOCH & HOUSEGO), with a slower acoustic velocity (v=820  m/s) and an effective aperture of $2\times 6\;{\mathrm{mm}}^{2}$ (H=2  mm), as the slow-axis scanner. Its transition time was ∼3  μs, with a maximum line scanning frequency of ∼80  kHz. In actual use, its maximum frequency was set to 10 kHz, at which speed no obvious astigmatism effects were detected, indicating that no additional compensation was required.

### 2.2 Two-Photon Imaging Setup

**Fig. 1:**
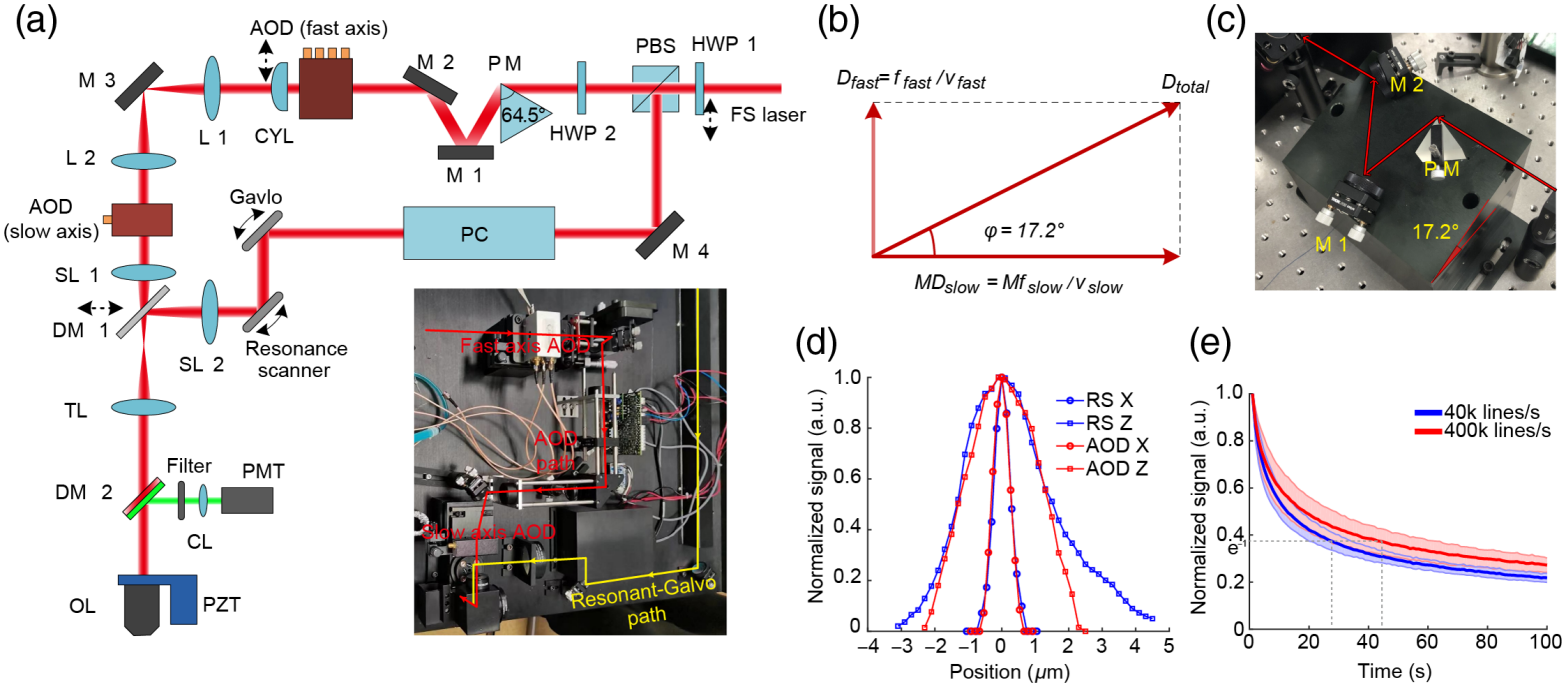
Principle of the setup. (a) Schematic diagram of the two-photon imaging setup. Bottom: A photograph showing the two paths of the laser scanning system. (b) A graph of the synthetic angular dispersion vector. (c) The dispersion compensating unit. (d) Normalized fluorescence intensity curves for the 0.51-μm bead along the X axis (circular) and Z axis (square) in RS mode (blue) and AOD mode (red). (e) The normalized photobleaching curves of 2-μm fluorescent beads at line scan frequencies of 40 kHz (blue) and 400 kHz (orange). Both curves are an average of eight measurements. The shadows are error bars.

A schematic diagram of the two-photon microscope used in this study is shown in Fig. 1(a), with a photograph that includes part of the laser scanning system shown at bottom. The device contained two parallel scanner paths: an AOD path for small subfield kHz imaging and an RS path for conventional video-rate full-field imaging. In this configuration, a laser produces a femtosecond beam with a central wavelength of 1040 nm (Origami-10, Onefive) or 920 nm (Mai Tai DeepSee, Spectra-Physics). A half-wave plate (HWP1) and a polarization beam splitter (PBS) are used to select the scanning path. A piezoelectric motor (ELL6K, Thorlabs) is used to switch the HWP1, then select whether the laser is transmitted or reflected at the PBS to enter one or the other scanning paths. In the AOD path, the femtosecond beam is expanded to 4 mm before hitting the dispersion compensating prism. A single prism (PM, custom-designed) is used to simultaneously compensate for the spatial and temporal dispersion of the two AODs.^29^ Another half wave plate (HWP2) is used to maximize prism efficiency. Upon exiting the prism, the beam is lifted into the scanning system for entry into the fast-axis AOD. A CYL compensates the astigmatism when the fast-axis AOD works with a chirped acoustic wave. A pair of doublet lenses (L1: F=200  mm, L2: F=100  mm) are used to conjugate the fast-axis and slow-axis AODs, changing the laser beam size from 4 to 2 mm, resulting in a beam expansion ratio (M) of 0.5 between the fast-axis and slow-axis AODs. Another pair of lenses (SL1: F=50  mm, TL: F=180  mm) are used to conjugate the AODs with the immersion objective lens (OL, 40×, NA=0.8, Nikon). The laser beam diameter expands to 7.2 mm to almost completely fill the back aperture of the objective. At the resonance path, a Pockels cell (PC) is used to adjust the laser power. A 12-kHz resonance scanner (CRS12K, Cambridge Technology) is used for fast-axis scanning and a Galvo (6215H, Cambridge Technology) is used for slow-axis scanning. Another scanning lens (SL2: F=100  mm) was added to form a 4f pair with the tube lens (TL) and a dichroic mirror (DM1) is used to manually switch scan paths. A piezoelectric actuator (PZT, MIROS500, Piezosystem Jena) drives the OL during axial scanning.

In the fluorescence detection path, a dichroic mirror (DM2, HC_735_LP, Semrock) splits the excitation laser and emission fluorescence, while an infrared cut-off filter (ET700SP-2P8, Chroma) further removes infrared light from the laser beam. Fluorescence signal is then collected by a collector lens (CL) and sent to a photomultiplier tube (PMT, H10770PA-40, Hamamatsu).

The electronic control system is based on the PXI platform (PXIe-1082, National Instruments). Two signal generator cards (PXIe-5413, National Instruments) are used to generate the amplitude and frequency modulation waveforms for custom-made voltage-controlled oscillators, which provide driving RF signals for the two AODs. A high-precision oscilloscope card (PXI-5122, National Instruments) records signals from the PMT, while an FPGA-based card (PXIe-7851R, National Instruments) generates the synchronization signal for signal generators and oscilloscopes. Custom-designed LabVIEW software is used for image acquisition, reconstruction, and preservation.

### 2.3 Dispersion and Astigmatism Compensation

AODs can introduce distortion through dispersion and astigmatism. Compensation is thus necessary to ensure that optical resolution is close to the diffraction limit. Because different AOD types were used for each of the scanning axes, they have different angular dispersion (D), i.e., the ratio of central ultrasound frequency (f) to the sound velocity (v) of the crystal. The beam expansion ratio (M) between the AODs can also affect the synthetic angular dispersion vector ($D_{\text{total}}$), as shown in Fig. 1(b). The size and direction of the synthesized angular dispersion can be calculated by Eqs. (3) and (4), respectively:

$$|D_{\text{total}}|=\sqrt{{\left|D_{\text{fast}}\right|}^{2}+{\left|MD_{\text{slow}}\right|}^{2}}=\sqrt{{\left(f_{\text{fast}}/v_{\text{fast}}\right)}^{2}+{\left(Mf_{\text{slow}}/v_{\text{slow}}\right)}^{2}},$$ (3)

$$\varphi =\arctan(|D_{\text{fast}}|/|MD_{\text{slow}}|)=\arctan(\frac{f_{\text{fast}}v_{\text{slow}}}{Mf_{\text{slow}}v_{\text{fast}}}).$$ (4)

Using M=0.5, $f_{\text{fast}}=115\;\mathrm{MHz}$, and $f_{\text{slow}}=145\;\mathrm{MHz}$, we calculate the size of synthesized angular dispersion as equal to $9.26\times 10^{-2}\;\mathrm{mrad}/\mathrm{nm}$, while the angle φ is equal to 17.2 deg. The prism design mainly considers the dispersion constant and ellipse ratio of the output beam. A custom MATLAB program was used to calculate the best apex angle and incidence angle, as previously described.^29^^,^^31^ Based on these calculations, we selected an SF11 prism with a 64.5-deg apex angle and 69.4-deg incidence angle that offers an angular dispersion of $9.26\times 10^{-2}\;\mathrm{mrad}/\mathrm{nm}$, an ellipse ratio of ∼1, and a theoretical efficiency of ∼95% at 1040 nm. A second SF11 prism with a 61.2-deg apex angle and 63.4-deg incidence angle was designed that could provide the same angular dispersion and ellipse ratio at 920 nm. We also designed a proprietary prism mounting module to ensure that the incident angle of the prism and the angle between the prism and the horizontal plane were both correct [Fig. 1(c)]. The distance between the prism and the fast-axis AOD was set to 120 cm, which offers approximately $-22,000\;{\mathrm{fs}}^{2}$ GDD to compensate for material dispersion in this configuration (AODs: $∼15,000\;{\mathrm{fs}}^{2}$, other optical components: $∼7000\;{\mathrm{fs}}^{2}$).

In addition to dispersion, astigmatism also requires compensation when the fast-axis AOD operates in fast scanning mode, during which the AOD performs like a CYL.^32^ The equivalent focal length of the CYL can be expressed as Eq. (5)

$$F_{\mathrm{CYL}}=\frac{v^{2}\Delta t}{\lambda \Delta f}.$$ (5)

At a line scan frequency is 400 kHz, line scan time (Δt) is 2.5  μs. If v=4200  m/s and Δf=50  MHz, then focal length of the compensating CYL is equal to 848 mm at 1040 or 958 mm at 920 nm. In addition to the 400-kHz line scan frequency, the fast-axis AOD could also be set to 40 kHz scanning frequency. However, at this lower frequency, the fast-axis AOD produces relatively negligible astigmatism and does not affect optical resolution. Another piezoelectric motor (ELL6K, Thorlabs) was used to add or remove the CYL from the optical path when the fast axis AOD is switched between different scanning frequencies.

To validate the effectiveness of compensation for dispersion and astigmatism, 0.51-μm fluorescent beads (Dragon Green, FC03F, Bangs Laboratories) were used to test the spatial resolution of this two-photon microscope. Normalized signal intensity curves for the bead along the X-, Y-, and Z-axes revealed full-width at half-maximum values of 0.60±0.05  μm (n=15) along the X axis (fast-axis), 0.56±0.04  μm (n=15) along the Y axis (slow-axis), 2.91±0.14  μm (n=15) along the Z axis in RS mode and 0.62±0.03  μm (n=15) along the X axis (fast-axis), 0.62±0.02  μm (n=15) along the Y axis (slow-axis), and 2.59±0.14  μm (n=7) along the Z axis [Fig. 1(d)]. This result indicated that dispersion and astigmatism in AOD mode were effectively compensated.

### 2.4 Imaging Mode and Parameter

Various imaging modes and scanning parameters were available for different application requirements, as shown in Table 1. The experimenter could flexibly and quickly switch between different scanning settings during one experiment according to the experimental design.

**Table 1:** Imaging modes and parameters.

Scan path	Fast-axis scanning frequency	Pixel size	Frame rate and image pixels
AOD mode	400 kHz	0.133 μm	10,000 Hz @ 250 × 40 pixels
			5000 Hz @ 250 × 80 pixels
			1600 Hz @ 250 × 250 pixels
		0.267 μm	10,000 Hz @ 125 × 40 pixels
			3200 Hz @ 125 × 125 pixels
			1600 Hz @ 125 × 250 pixels
		0.665 μm	10,000 Hz @ 50 × 40 pixels
			3200 Hz @ 50 × 125 pixels
			1600 Hz @ 50 × 250 pixels
		1.33 μm	3200 Hz @ 25 × 125 pixels
	40 kHz	0.133 μm	1000 Hz @ 250 × 40 pixels
			500 Hz @ 250 × 80 pixels
			160 Hz @ 250 × 250 pixels
RS modes	24 kHz (bidirection)	0.05 to 1 μm (continuously adjustable)	40 Hz @ 512 × 512 pixels
			20 Hz @ 1024 × 1024 pixels

### 2.5 Increasing Line Scan Frequency Reduces Rate of Photobleaching

Next, we measured photobleaching rates at 40k and 400k line/s scan frequencies. For this experiment, 2-μm fluorescent beads (Flash Red, FSFR005, Bangs Laboratories) were continuously imaged over a 100-s interval to produce obvious bleaching. The average laser power under the OL was set to 13.6 mW for both frequencies. Normalized curves of signal intensity during photobleaching showed higher photobleaching under 40 kHz scanning than 400 kHz [Fig. 1(e)]. Notably, the rate of photobleaching decreased with higher line scan frequency. Comparison of bleaching rates based on time until fluorescence intensity decay to 1/e (36.8%) showed that, at 40 kHz, this decay time was 28.8 s, but 45.8 s at 400 kHz, indicating that signal intensity persisted ∼59% longer at the higher frequency.

These results were consistent with previous studies^20^^,^^33^ that showed the formation of triplet states is increased under a prolonged, single, continuous illumination time, which ultimately accelerates the photobleaching rate. Taken together, these assays demonstrate that increasing line scan frequency to hundreds of kilohertz can be beneficial for reducing photobleaching rates.

### 2.6 Animal Setup

C57BL/6J male mice (3-months old) were obtained from the Laboratory Animal Center at the Army Medical University. Thy1-GFP mice were purchased from the Jackson Lab as JAX# 011070. Mice were socially housed in their home cages under a 12-h light/dark cycle (lights on at 7 a.m.). All experimental procedures were carried out according to the animal ethical guidelines and were approved by the Third Military Medical University Animal Care and Use Committee.

#### 2.6.1 Virus injection

A chronic cranial window was prepared for ultrafast imaging. Mice were prepared sterilely as described in a previous study.^9^ In brief, mice were anesthetized in 1% isoflurane (pure oxygen, 500  ml/min) and placed in stereotactic apparatus. To maintain the health of the imaging cortex, the inclining virus injection method was used. A craniotomy (∼0.5  mm in diameter) was performed above the dorsal auditory cortex [anteroposterior (AP): −3.1  mm, mediolateral (ML): −3.8  mm, related to bregma]. Filling with the AAV2/8-hsyn-GCaMP6f virus (∼80  nl, $∼1\times 10^{13}\;\mathrm{vg}/\mathrm{ml}$, Shanghai Heyuan Technology Corp., Ltd., China), the inclined electrode (diameter: ∼20  μm, angle: 65 deg to the horizontal) was moved forward 1.4 mm to reach the target layer 2/3 of the auditory cortex (AuC) (∼0.3  mm depth from pia) with slow velocity (20  nl/min). Before retracting, a 5-min steady time was used for virus diffusion. Prophylactic injections of antibiotics (Cefazolin, 500  mg/kg, North China Pharmaceutical Group Corporation, China) were given intraperitoneal injection twice per day for 3 days after surgery. Two-photon imaging was performed after 14 days of virus expression.

#### 2.6.2 Cranial window setup

The custom chamber was sealed to the skull with dental acrylic (Superbond C&B, Sun Medical, Japan). A craniotomy (2.5 mm in diameter) was made over the left AuC after the dental acrylic concretionary. The surrounding bone of the cranial window was polished to an optimal size for embedding the coverslip. The coverslip was embedded and sealed with dental acrylic (Tetric EvoFlow T1). Prophylactic injections of antibiotics (Cefazolin, 500  mg/kg, North China Pharmaceutical Group Corporation, China) were given intraperitoneal injection twice per day for 3 days after surgery.

#### 2.6.3 Single cell electroporation

Same as our previous studies, single cell electroporation was used for labeling the dendrites and spines of AuC neurons. In brief, “shadow imaging” technique was performed to target the neurons of interest by a borosilicate electrode (12 to 15  MΩ) filled with OGB-1 $6K^{+}$ (2.5 mM, Oregon Green 488 Bapta-1 potassium salt, Molecular Probes). Once the tip of the electrode attached the membrane of the neuron, the OGB-1 $6K^{+}$ was loaded into neurons by 100 rectangular pulses (−10  V, 0.5 ms duration at 50 Hz). The pulses for electroporation were generated with a series of stimuli triggered by a Master-8 (A.M.P.I, Israel) and were delivered to the neuron by the MVCS-02 iontophoresis system (NPI Electronic).

#### 2.6.4 Blood vessel labeling

At first, the head-fixed GFP-transgenic mice were performed cranial window implantation in AuC (for details see Sec. 2.6.2). With a 7-day recovery after cranial window implantation, the mice were anesthetized in 1% isoflurane (pure oxygen, 500  ml/min) and placed in stereotactic apparatus. To image blood vessel, blood serum was labeled 0.1 ml bolus of 5% (w/v) Texas Red dextran (70 kDa) in PBS through a tail-vein injection. The hemodynamic imaging was last as long as ∼2  h.

#### 2.6.5 In vivo two-photon imaging

All the two-photon imaging were performed on head-fixed mice. For calcium imaging or hemodynamic imaging, we adopt a two-step zoomed-in imaging method. First, large-scale imaging was performed through RS scanning to determine the targeted region to be further imaged. Then, by switching to AOD scanning mode, the target area was sampled with high time resolution. In this mode, a variety of ultrafast imaging modes were performed in many scenarios. The two-photon imaging lasted as long as ∼2  h. Laser power was measured below the objective in different scenes: 20 to 30 mW in GFP imaging or Texas Red dextran labeling hemodynamic imaging, 20 to 40 mW in OGB-1 or GCaMP labeling calcium imaging, ∼40  mW in autofluorescence hemodynamic imaging.

### 2.7 Data Analysis

Data analysis was performed with custom-written software using LabVIEW 2017 (National Instruments), Igor Pro 6.0 (Wavemetrics), Image J (NIH), Prism 8.4 (GraphPad), and MATLAB 2018b (Mathworks).

To batch calibrate the brain motion (x−y motion) along the imaging focal plane caused by animal breath and heartbeat, we used customized correction software (MATLAB 2018b, MathWorks). The correction code is based on the image alignment software TurboReg (ImageJ, NIH). Relative fluorescence changes (Δf/f) were used in most ${\mathrm{Ca}}^{2+}$ response data in this paper. $\Delta f/f=(f-f_{0})/f_{0}$ were calculated as ${\mathrm{Ca}}^{2+}$ signals, where the baseline fluorescence $f_{0}$ was estimated as the 25th percentile of the entire fluorescence recording. The comparison data from unpaired groups were used by Wilcoxon rank-sum test, and the comparison data among multiple groups were used by one-way ANOVA test.

**Fig. 2:**
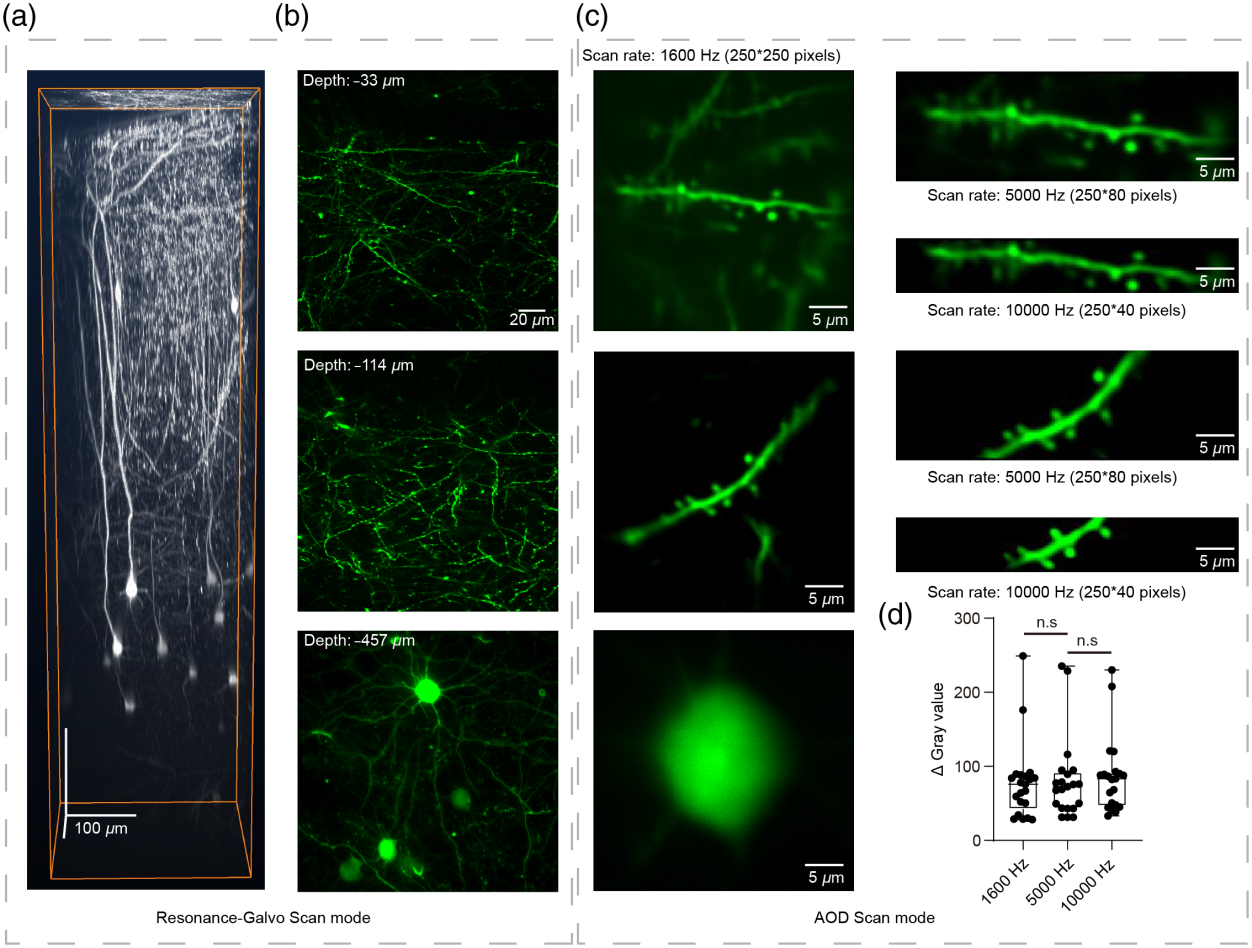
Demonstration of RS and AOD imaging: multiscale morphology of neuron sampled by RS and AOD scan modes. (a) 3D morphology reconstruction of a column of AuC in a Thy1-GFP transgenic mouse (250×250×700  μm). (b) Fields of view from the column in (a) at different depths recorded by RS mode. (c) Representative images of the three scan modes (1600, 5000, and 10,000 Hz). (d) Comparison of ΔGray values from a 10-s scan in each AOD mode, 1600 versus 5000 Hz, p=0.9687; 1600 versus 10,000 Hz, p=0.815. 21 dendritic shafts in five focal planes, One-way ANOVA test.

In Fig. 2(d), normalized ΔGray value was performed for comparison of the capacity of detecting the fluorescence among different scan modes. First, the 1-s sampling averaged photos of different scan modes were performed background normalizing by software Background Correction (ImageJ, NIH). Second, the same line segment of fixed location was penetrated to the same component of dendrite. Third, the normalized ΔGray value was detected by software Histogram (ImageJ, NIH).

**Fig. 3:**
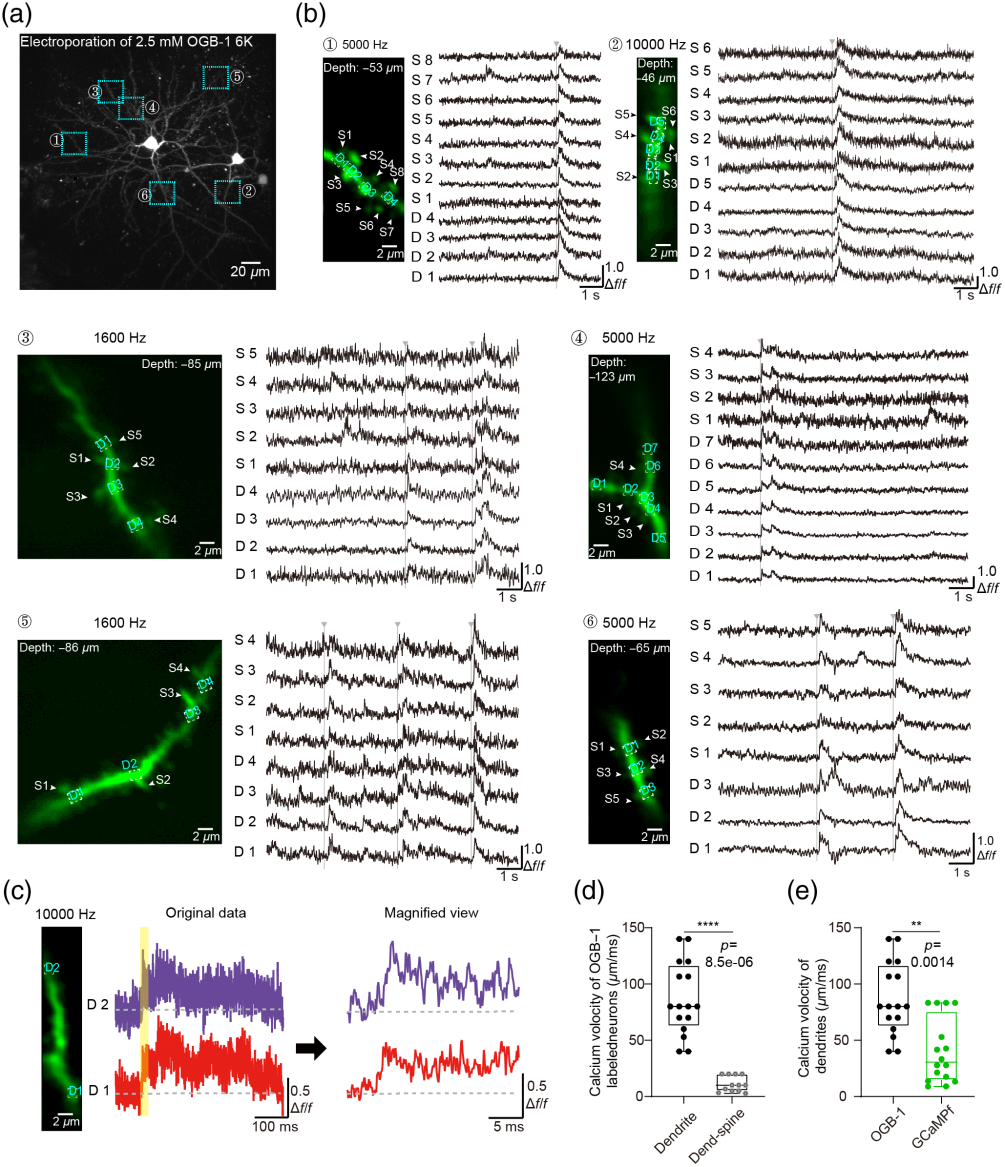
Calcium imaging by RS and AOD scan from population level to subcellular level. (a) Z-stack reconstruction of an OGB-1-labeled neuron in AuC (300×300  μm, recorded by RS scan mode). Regions in rectangles 1 to 6 on each dendritic segment were selected for further scanning. (b) Magnified view of calcium signals in each region of (a) recorded with AOD scan mode, the inverted gray arrow lines indicated the spontaneous propagation events. (c) (Left) An example two-photon image of a dendritic segment (averaged 10,000 frames). (Middle) Raw calcium signal data from the two points (D1 and D2) on the dendritic shaft (10,000 Hz). (Right) Magnified view of calcium signal data for the D1 and D2 points on the dendritic shaft from the yellow shaded region in the raw data. (d) Comparison of calcium velocity of dendritic propagation and dendritic shaft region—spine of OGB-1-labeled neuron. ****, P=8.5e−06, n=16 dendritic shaft events, n=12 dendritic shaft region–spine events, in five neurons. Wilcoxon rank sum test. (e) Comparison of dendritic calcium propagation velocity between OGB-1-labeled neurons and GCaMP6f-labeled neurons. **, P=0.0014, n=16 events out of five OGB-1-labeled neurons, n=16 events out of four GCaMP6f-labeled neurons. Wilcoxon rank sum test.

In Fig. 3, cross-covariance analysis was applied for two ROIs of time series of calcium propagation speed calculation. The cross covariance $r_{xy}$ Eq. (6) as

$$r_{xy}[L]=\frac{1}{N}\overset{N-L}{\underset{n=1}{\sum }}(x_{n+L}-\overset{¯}{x})(y_{n}-\overset{¯}{y}),$$ (6)

where x and y represent two ROI series, N is the total number of frames in the ROI series, $\bar{x}$ and $\overset{¯}{y}$ are the average of the two ROI series. Find the L value when the $r_{xy}[L]$ is the maximum value. If L is positive value, the x(t) has to precede the y(t); if L is negative value, the y(t) has to precede the x(t). The time difference between the two is L×Δt. Δt is the time of a frame, the reciprocal of the frame rate.

**Fig. 4:**
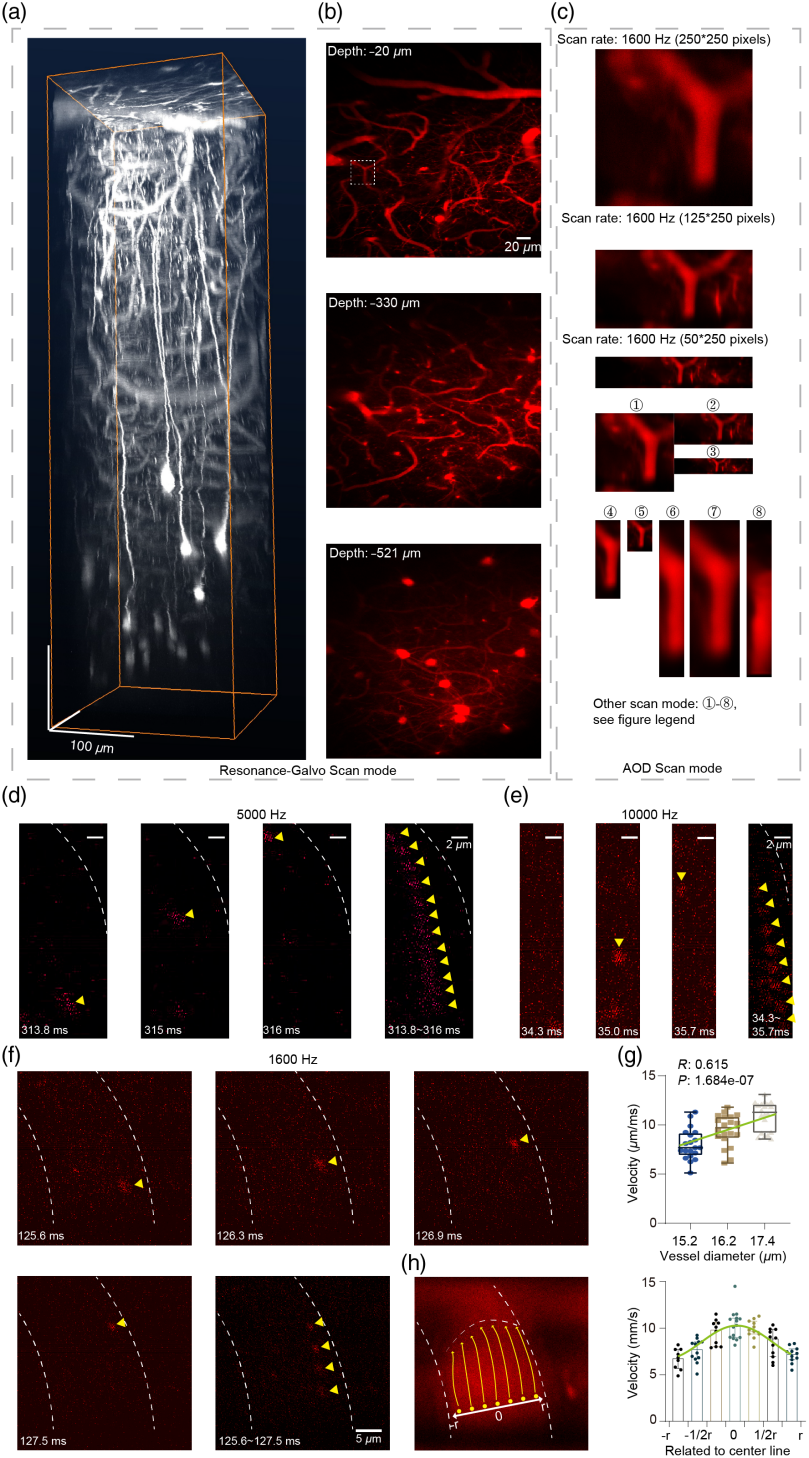
Demonstration of RS and AOD hemodynamic imaging: multiscale morphology of blood vessels sampled by RS scan mode and AOD scan mode. (a) 3D reconstruction of vascular morphology in a column (250×250×700  μm) of AuC in a GFP-labeled mouse. Blood vessels were stained with Texas Red Dextran. (b) Fields of view at different depths in the column in (a) were scanned in RS mode. (c) Magnified view of the representative blood vessel from the white dashed region in (b). Representative images collected with 11 different scan modes are shown. Scan rates for modes 1 to 8: 3200 Hz (125×125  pixels), 3200 Hz (50×125  pixels), 3200 Hz (25×250  pixels), 10,000 Hz (125×40  pixels), 10,000 Hz (50×40  pixels), 10,000 Hz (250×40  pixels), 5000 Hz (250×80  pixels), and 1000 Hz (250×40  pixels). (d) Hemodynamic signals of an example blood vessel sampled by 5000 Hz scan (Video S5, MP4, 9.42 MB [URL: https://doi.org/10.1117/1.NPh.10.2.025006.s5]). Representative images of an RBC within a single frame sequentially collected at 313.8, 315, and 316 ms of a 1-s scan. Combining the 12 total images collected from 313.8 to 316 ms (right) shows full trajectory. Yellow arrowheads indicate RBC location. Scale bar: 2  μm. (e), (f) The same image sampling as in (d) collected by scanning at 10,000 Hz in (e) (Video S6, MP4, 5.29 MB https://doi.org/10.1117/1.NPh.10.2.025006.s6) and 1600 Hz in (f) (Video S4, MP4, 9.60 MB https://doi.org/10.1117/1.NPh.10.2.025006.s4). (g) Comparison of RBC velocity in three different diameter vessels. N=20 RBCs per vessel. (h) Hemodynamic simulation of RBC trajectory in the 15.2-μm vessel in (g). (Left) The curve at the head of the yellow arrows represents differences in blood flow velocity at different positions within the vessel. (Right) Histogram of RBC velocity at different positions relative to the vessel center. A fitting curve is presented at the top of the columns.

For velocity calculation and fitting of blood flow in Fig. 4, the blood cells detected were divided into seven parts according to their locations related to the center line. The line-shape region of the RBC trajectory was marked along the blood vessel by ImageJ. The length of the RBC trajectory was traced out and certain velocities were equal to the length divided by the time from the first frame to the last frame. For the diameter calculation of blood vessels, the average image of the full-frame data was generated by ImageJ. Then five orthogonal lines to the long axis of the blood vessel were first extracted, which were evenly distributed in the head and tail of blood vessels. The mean value of the five orthogonal lines was considered to be the blood vessel diameter. Finally, according to the different velocities of the RBCs’ locations, a quadratic function was fitted by MATLAB (MathWorks).

## 3 Results

### 3.1 Morphology of Neurons

At the beginning, RS and AOD two-photon microscopy was used to image neuron morphology in AuC of Thy1-GFP transgenic mice. To explore its capacity for imaging at a depth of several hundred microns *in vivo* [Figs. 2(a) and 2(b)], three-dimensional (3D) scanning imaging was initially conducted in RS mode. After examining XZ maximum projection of the 3D images [Fig. 2(a)], two dendritic regions and a neuron soma [Fig. 2(b)] were selected for further imaging by AOD-based scanning with different scan modes (1600, 5000, and 10,000 Hz) [Fig. 2(c)]. No differences were detected in the normalized ΔGray value among the three scan modes using the same scan time [Fig. 2(d)]. These results indicated that, after dispersion and astigmatism compensation, the spatial resolution of this system was sufficient to image neural dendrites and dendritic spines in fast imaging mode *in vivo*.

### 3.2 Calcium Signal Propagation

In this study, two different calcium indicators (i.e., a synthetic chemical ${\mathrm{Ca}}^{2+}$ dye, OGB-1 and a genetically encoded ${\mathrm{Ca}}^{2+}$-sensitive protein, GCaMP6f) were used to label single cells for studying ${\mathrm{Ca}}^{2+}$ signal propagation (Fig. 3). A z-stack of images of an OGB-1-labeled neuron was obtained using RS mode [Fig. 3(a)]. To observe the calcium signals of dendritic spines and their corresponding shafts, a subset of dendritic regions was imaged by AOD scanning [Fig. 3(b)], which were magnified from different dendritic shafts in the z-stack [insets 1 to 6 in Fig. 3(a)]. In addition, ultrafast imaging of GCaMP6f-labeled neurons was also performed in AuC in awake mice (Fig. S1 in the Supplemental Material). The GCaMP6f calcium signals were captured in soma by three AOD scan modes [Fig. S1(a) in the Supplemental Material], as well as these in a dendritic shaft [Fig. S1(b) in the Supplemental Material]. The spontaneous calcium propagation signals were obtained in a dendritic shaft of OGB-1-labeled neuron [Fig. 3(c)], and it revealed an extremely tiny difference in calcium propagation between the furthest points on the dendritic shaft. Using cross-correlation analysis of different sites on the dendritic shafts (See Sec. 2.7), calcium signal propagation measurements between spines and their corresponding dendritic shafts indicated velocities of 10/5–20  μm/ms, which were markedly slower than that in dendrites [Fig. 3(d)]. By comparison, the calcium propagated velocity of the dendritic shaft in GCaMP6f-labeled neurons (30/14.8–75.43  μm/ms; n=16 events from four neurons) was considerably slower than that of OGB-1-labeled neurons (80/62.5–116.7  μm/ms; n=16 events from five neurons) [Fig. 3(e)].

### 3.3 Hemodynamic Characterization at Single Cell Resolution

To benchmark the sensitivity of velocity measurement *in vivo*, we observed the movement of single RBCs *in vivo*. To this end, 3D imaging by RS scanning was conducted in an anesthetized, GFP-transgenic mouse [Fig. 4(a)] with blood vessels labeled by Texas Red Dextran staining. After examining the 3D scanning images at different depths [Fig. 4(b)], a representative vessel was selected for subsequent imaging by AOD-based scanning using 11 scan modes [Fig. 4(c)]. Hemodynamic imaging of the blood vessel labeled Texas Red dextran was performed in awake mice (Fig. S2 in the Supplemental Material; see also Videos S1, S2, and S3). The results show that the flow rate of RBCs in capillary vessels with a diameter of 5  μm is 1.35±0.15 (mean ± SEM) mm/s, which is consistent with previous studies.^34^^,^^35^ Moreover, we also demonstrated that 920-nm femtosecond laser could excite the autofluorescence signal of RBCs on the blood smear *in vitro* (see Fig. S3 in the Supplemental Material). Although the autofluorescence signal is weak, its brightness is enough for us to trace the flow of RBCs (see Videos S4, S5, and S6). Hemodynamic signals were recorded for 1 s duration in vessels of different diameters by autofluorescence imaging without labeling [Figs. 4(d)–4(h)]. In one example, at 313.8, 315, and 316 ms of sampling, the trajectory of the same blood cell was detected at sequential locations in a blood vessel [Fig. 4(d)]. Hemodynamic signals were also detected by scanning at 10,000 [Fig. 4(e)] and 1600 Hz [Fig. 4(f)]. Blood flow velocity through vessels of different diameters (N=20 RBCs per vessel, three vessels) were quantified, and the movement velocity of RBCs was as 9.466±0.25  mm/s [Fig. 4(g)]. Linear correlation analysis indicated that flow velocity was positively correlated with vessel diameter (N=20 RBCs per vessel, three vessels, R=0.615, P=1.684e−07). Noted that the velocity of blood flow differed between positions in the vessel section [Fig. 4(h)], with higher velocity associated with the central axis compared with that near the vessel wall. The velocity dependency on radial distance followed a parabola fitting curve ($r^{2}=0.9477$), which is basically predicated by Poiseuille’s law of laminar flow. These results thus demonstrated the capture of a blood cell trajectory and velocity estimation through the high temporal resolution provided by ultrafast frame-scanning two-photon imaging.

## 4 Discussion

In this study, we used AODs to further increase the frame rate of full-frame 2D two-photon imaging. More specifically, this system adopted a high acoustic velocity AOD as the fast-axis scanner, with a transit time of ∼1  μs, which significantly increased the line scan frequency to 400 kHz, ∼5 times higher than that developed in previous studies.^20^ To maximize the frame rate, another AOD was used as the slow axis scanner to achieve a maximum frame rate of 10,000 Hz, which was ∼3 times faster than the maximum effective scanning rate of Galvo mirrors.^20^^–^^25^ While improving the frame rate, both the spatial and temporal pixel sampling rate still met the oversampling conditions to improve the signal accuracy. The bandwidth of the photo-current signal was limited to ∼40  MHz after the electronic amplifier, and the pixel sampling rate was 100 MHz, with ∼2.5 times of oversampling; The minimum pixel size was 0.133  μm, and the optical resolution was 0.62  μm, with ∼4.7 times of oversampling. Oversampling facilitated imaging of small neurite structure with weak fluorescence, such as dendritic spines.

In this configuration, the AOD scanning path was integrated with a parallel resonant-Galvo path, although these components could also be combined in a serial configuration. We chose the parallel configuration because of the higher laser transmittance required by both paths. The fast-axis AOD enhanced the scanning frequency through high acoustic velocity, but correspondingly resulted in fewer resolution spots thus effectively a smaller field of view for images. This shortcoming was complemented by the resonant-Galvo component, which provided a larger field of view with sufficient resolution power of diffraction-limited spots.

In two-photon imaging, AODs can function in either 2D full-frame scanning mode or in random-access mode.^14^^–^^18^ AOD-based 2D or 3D random-access microscopy can simultaneously sample the footprint of hundreds of cells at submillisecond scale time resolution.^14^^–^^18^ In contrast, imaging by full-frame scanning enables not only complete optical sampling of entire cells in focal plane but also more effective determination of the impacts for applying robust algorithms to reduce signal artifacts introduced by animal movements. Nevertheless, the two scanning modes are not incompatible. Our method described in this study could be further combined with a random-scanning mode by electrically switching the spot diameter incident on the AODs and the beam expansion ratio between the AODs and the OL. When working in random-scanning mode, the laser diameter should also be increased to fill the AOD to maximize the field of view in images. However, when working in full-frame scanning mode, the laser diameter should be reduced to accommodate a ∼1-μs transit time while the line scan frequency is increased to hundreds of kilohertz.

Electrical and calcium signal propagation within the dendritic tree of a neuron play vital roles in single-neuron computation, learning, and plasticity *in vivo*. Due to technical limitations, most quantitative studies that measured such rapid signal propagations took place in *in vitro* preparations,^36^^,^^37^ see also Table S1 in the Supplemental Material). Overall, direct electrical measurement of voltage propagation shows the highest propagation velocity above 500  μm/ms.^37^ However, such measurements can be hardly performed *in vivo*. Notably, the propagation velocity measured with genetically encoded ${\mathrm{Ca}}^{2+}$ indicators measurements of GCaMP6s-expressing cultured neurons. (Ref. 22, ∼0.025  μm/ms) are much (almost four order of magnitude) lower than that measured with small-molecule ${\mathrm{Ca}}^{2+}$ dye *in vitro* (Ref. 17, 227±14  μm/ms), which is likely not due to experimental conditions but due to the differences in binding kinetics of the ${\mathrm{Ca}}^{2+}$ indicators. Consistently, here in this study, the ${\mathrm{Ca}}^{2+}$ propagation velocity measured with OGB1-6K dye (80/62.5–116.7  μm/ms) was also much higher that measured with GCaMP6f (30/14.8–75.43  μm/ms). Thus, the higher framerate (in the range of 10k instead of 1k) is required for *in-vivo* studies that needs to address the ${\mathrm{Ca}}^{2+}$ propagation direction and velocity which shall not be confounded by the slow kinetics of genetically expressed ${\mathrm{Ca}}^{2+}$ indicators.

## 5 Conclusion

Here, we describe a new high-speed two-photon microscope that incorporates a high acoustic velocity AOD. This microscope has a maximum frame rate of 10,000 fps at 250×40  pixels. To compensate for the small field of view in high-speed AOD scanning, we introduced a parallel RS scanning component that enables two-photon imaging from the population level to the subcellular level. As a proof-of-concept demonstration, we used this microscope to detect blood flow velocity (9.241  mm/s in a 16.3-μm diameter vessel) and dendritic calcium signal propagation velocity (80/62.5–116.7  μm/ms). In addition, our results show that photobleaching rates are reduced by increasing line scan frequency. Collectively, this study illustrates how our ultrafast AOD and RS two-photon microscope is a powerful tool for precise recording neural activities and hemodynamics *in vivo*, with high temporal resolution and high flexibility of several imaging modes.

## 6 Appendix

Captions and links are provided here for the supplemental videos.

Video S1: An example video of blood vessel labeled Texas Red dextran, which is slowed down to 160 fps from 1600 fps. https://doi.org/10.1117/1.NPh.10.2.025006.s1

Video S2: An example video of blood vessel labeled Texas Red dextran, which is slowed down to 100 fps from 5000 fps. https://doi.org/10.1117/1.NPh.10.2.025006.s2

Video S3: An example video of blood vessel labeled Texas Red dextran, which is slowed down to 100 fps from 10,000 fps. https://doi.org/10.1117/1.NPh.10.2.025006.s3

Video S4: An example video of blood vessel of autofluorescence without labeling, which is slowed down to 160 fps from 1600 fps. https://doi.org/10.1117/1.NPh.10.2.025006.s4

Video S5: An example video of blood vessel of autofluorescence without labeling, which is slowed down to 100 fps from 5000 fps. https://doi.org/10.1117/1.NPh.10.2.025006.s5

Video S6: An example video of blood vessel autofluorescence without labeling, which is slowed down to 100 fps from 10,000 fps. https://doi.org/10.1117/1.NPh.10.2.025006.s6

## Code, Data, and Materials Availability

Custom MATLAB code used in this report is available upon request by contacting Dr. Zhenqiao Zhou (zhouzq@sibet.ac.cn).
