# Supplementary material for: Ten-kilohertz two-photon microscopy imaging of single-cell dendritic activity and hemodynamics in vivo
Source: Neurophotonics. 2023 May 3;10(2):025006. doi: 10.1117/1.NPh.10.2.025006 (PMC10156610; doi:10.1117/1.NPh.10.2.025006)
Supplement: Supplementary file 1 [file NPh_010_025006_SD001.pdf]

Supplemental material

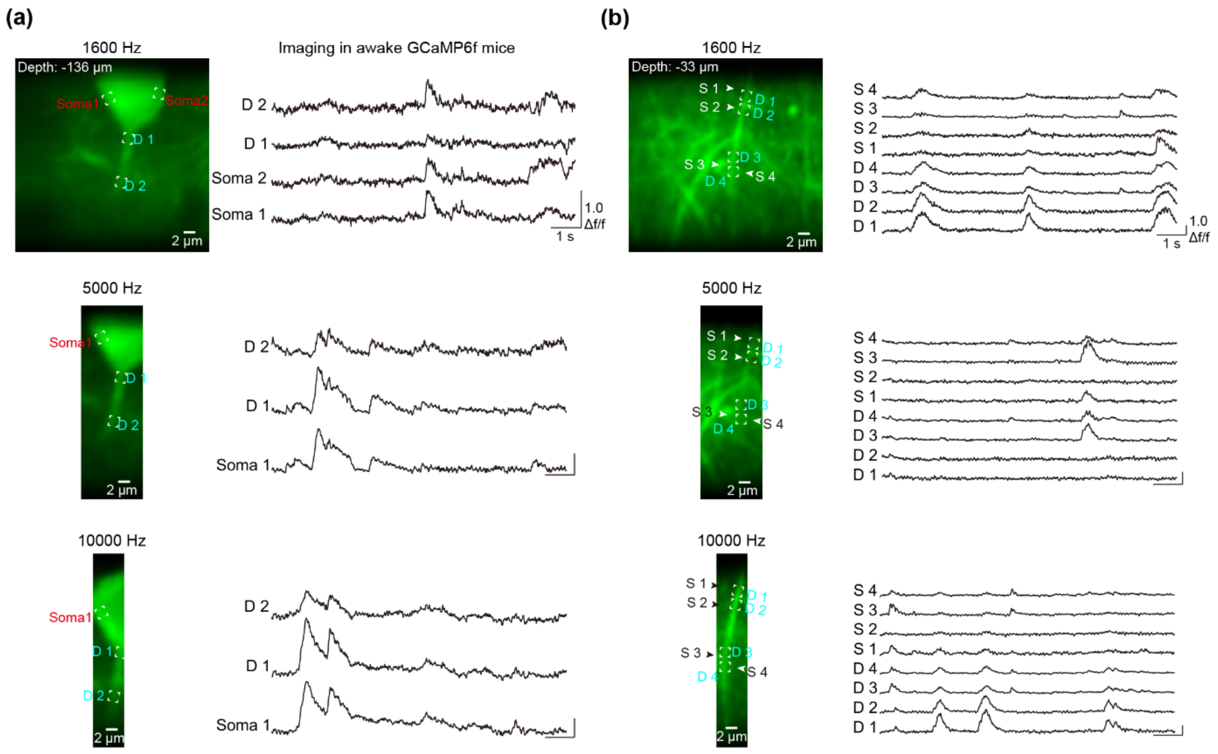

Figure S1. The calcium imaging of GCaMP6f labeled neuron by AOD scan. (a) Two photon imaging in the soma of GCaMP6f labeled neuron in awake mice. Upper to lower: The averaged two photon images and calcium signals of 3 scan modes were shown (1600 Hz, 5000 Hz, and 10000 Hz). (b) Same arrangement of the dendritic shaft of GCaMP6f labeled neuron in AuC as panel a.

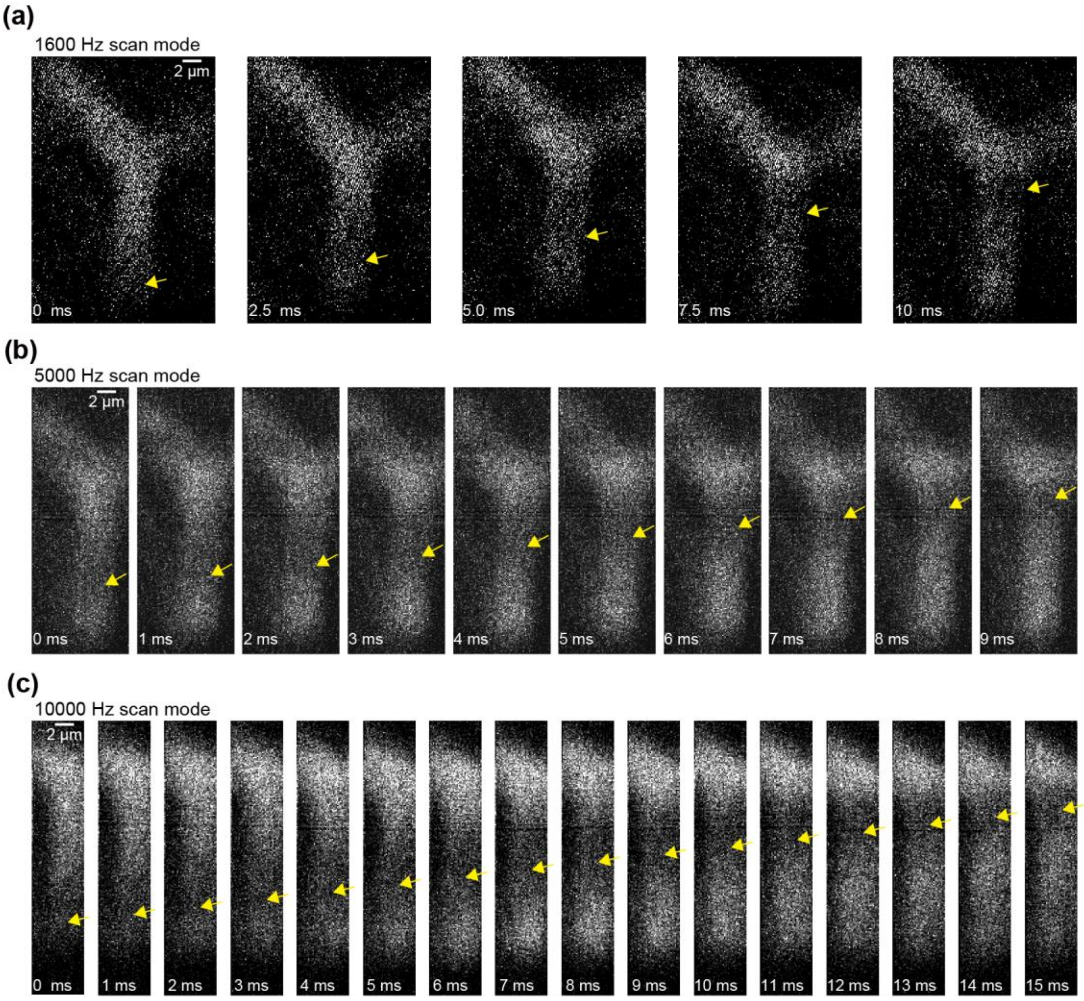

Figure S2. Ultrafast hemodynamic imaging of the blood vessel labeled Texas Red dextran. (a) Individual frames imaged at a 2.5 ms interval by 1600 Hz scan mode. (b) Individual frames imaged at a 1 ms interval by 5000 Hz scan mode. (c) Individual frames imaged at a 1 ms interval by 10000 Hz scan mode.

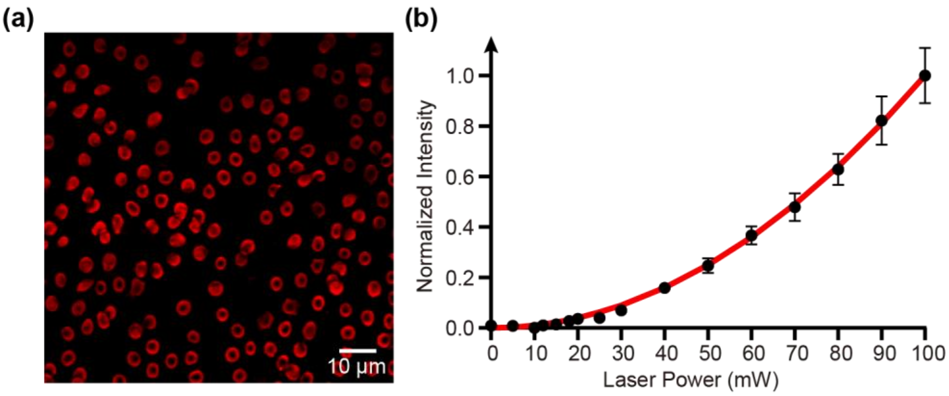

Figure S3. Autofluorescence of red blood cells under two-photon microscope. (a) Morphology of RBC on the blood smear in vitro. (b) The relationship between the normalized intensity and the power of 920 nm femtosecond laser.

Table S1. Overview of propagation velocity of dendrites

**Year**	**Author**	**Measuring object**	**Measurement mode**	**Propagation velocity**
1994	Stuart and Hauser	Purkinje cells of rat *in-vitro*	Double patch recording	240 μm/ms
1996	Larkum et al.	Motor neurons of rat spinal cord *in-vitro*	Double patch recording	500 μm/ms
2012	Katona et. al	Fluo-5F labeled pyramidal neurons *in-vitro*	Random-access AOD 2-photon imaging	227 ± 14 μm/ms
2020	Wu et. al	GCaMP6-expressing cultured neurons	kHz full-frame 2-photon imaging	0.025 μm/s
